# Florid cemento-osseous dysplasia: Report of a case documented with clinical, radiographic, biochemical and histological findings

**DOI:** 10.4317/jced.50854

**Published:** 2013-02-01

**Authors:** Harika Kutluay Köklü, Dilek A. Çankal, Süleyman Bozkaya, Gülfem Ergün, Emre Bar??

**Affiliations:** 1 Department of Oral and Maxillofacial Surgery, Faculty of Dentistry, Gazi University, Ankara, Turkey; 2 Department of Prosthodontics, Faculty of Dentistry, Gazi University, Ankara, Turkey; 3 Department of Oral Pathology, Faculty of Dentistry, Gazi University, Ankara, Turkey

## Abstract

Florid cemento-osseous dysplasia (FCOD) has been described as a condition that characteristically affects the jaws of middle-aged black women. This condition has also been classified as gigantiform cementoma, chronic sclerosing osteomyelitis, sclerosing osteitis, multiple estenosis and sclerotic cemental masses. It usually exhibits as multiple radiopaque cementum-like masses distributed throughout the jaws. Radiographically, FCOD appears as dense, lobulated masses, often symmetrically located in various regions of the jaws. Computed tomography, because of its ability to give axial, sagittal, and frontal views, is useful in the evaluation of these lesions. This article reports the case of a 45-year-old white man who was diagnosed with FCOD on the basis of clinical, radiographic, biochemical and histological findings. It is of major importance to realize that all dentists have a unique opportunity as well as ethical obligation to assist in the struggle against wrong dental treatments that might save patients dental health. This case report illustrates the point that periapical radiolucencies may represent benign fibro-osseous lesions that may be overlooked or result in unnecessary endodontic treatment.

** Key words:**Florid cemento-osseous dysplasia, florid osseous dysplasia, fibro-osseous lesions.

## Introduction

The classification of cemento-osseous lesions of the jaws has long been a complex and controversial dilemma for pathologists and clinicians. The current classification of cementomatous lesions, released in 1992 by the World Health Organization is based on age, sex and histopathologic, radiographic and clinical characteristics, as well as location of the lesion. This classification includes cemento-ossifying fibroma, benign cementoblastoma and the cemento-osseous dysplasia group, in which periapical cemental dysplasia and florid cemento-osseous dysplasia (FCOD) are included (1).

FCOD is a very rare condition presenting in the jaws. These lesions are most commonly seen in middle-aged black women, although it also may occur in Caucasians and Asians, and have been entitled as sclerosing osteitis, multiple enostoses, diffuse chronic osteomyelitis and gigantiform cementoma (2).

The process may be totally asymptomatic and, in such cases, the lesion is detected when radiographs are taken for some other purposes. Symptoms such as dull pain or drainage are almost always associated with exposure of sclerotic calcified masses in the oral cavity. This may occur as the result of progressive alveolar atrophy under a denture or after extraction of teeth in the affected area (3).

Radiographically, FCOD appears as dense, lobulated masses, often symmetrically located in two or more qua-drants, usually in the tooth-bearing regions. They are often confined within the alveolar bone (1,4).

Computed tomography (CT), because of its ability to give axial, sagittal, and frontal views, is useful in the evaluation of these lesions (4). Histologically, these lesions are composed of anastomosing bone trabeculae and layers of cementum-like calcifications embedded in a fibroblastic background (1,5).

Management of these conditions involves clinical radiographic follow-up. Endodontic therapy should not be done before a definitive diagnosis is obtained, especially when it is based solely on radiographic findings and no other signs and symptoms are present (6).

This article reports the case of a patient who was diagnosed with FCOD on the basis of clinical, radiographic, biochemical and histological findings.

## Case Report

**Figure 1:**
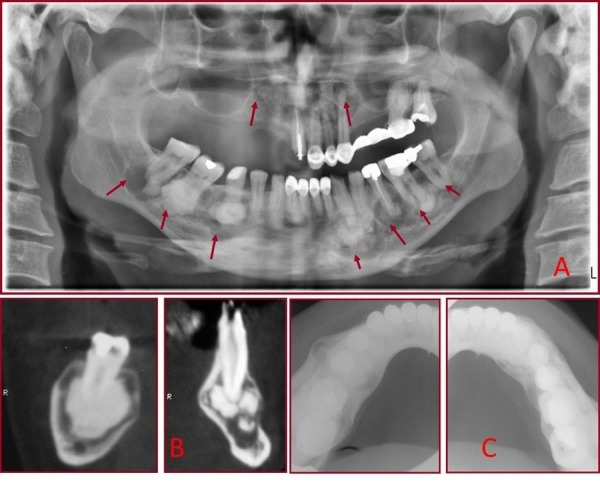
A. Panoramic radiograph showing multiple mostly dense mixed radiodense/radiolucent lesions closely associated with the roots of maxillary and mandibular teeth. Note that the epicenter of the lesions is above the inferior alveolar canal; B. Vertical reconstruction at level of the lower right second premolar and first molar region; C. Occlusal radiograph showing slight buccal expansion at the molar region in the right and left side of the mandible.

**Figure 2:**
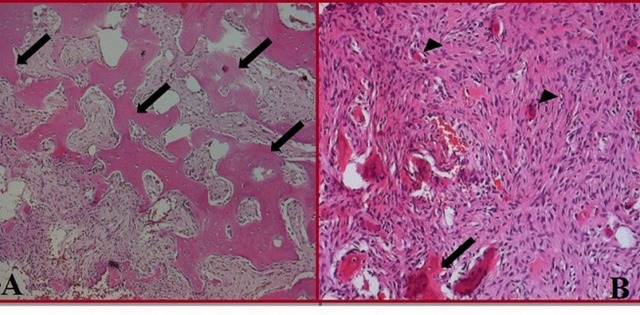
The lesion showed woven bone trabeculae (arrow) and cementum-like mineralization (arrowhead) within cellulary fibrous connective tissue (Hematoxylin-Eosin A:x100, B:x200).

In February 2010, a 45-year-old white man was referred to the Oral and Maxillofacial Surgery Department of the Dental Faculty of Gazi University for further investigation and treatment of his existing lesions. His medical history was non-contributory and extraoral examination was within normal limits. The intraoral examination revealed considerable expansion at the right and left posterior vestibular area of his mandible. The overlying gingiva and mucosa in mandible were normal without any clinical signs of inflammation. All teeth had normal vitality. There was no mandibular nerve paresthesia or facial deformation. Orthopantomograph and computed tomography (Fig. 1) showed multiple mostly dense mixed radiodense/radiolucent lesions closely associated with the roots of the upper left lateral incisor and canine, the lower left canine, premolars, first and second molars, the lower right second premolar, first and second molars. There was no root resorption or fusion of the lesions to the involved teeth. The occlusal images revealed slight buccal expansion at the molar region on the left and right side of the mandible (Fig. 1). Intraoral incisional biopsy was performed. Biopsy specimen of this lesion composed of benign fibro-osseous tissue containing bone which ranges from woven bone trabeculae to cementum like mineralizations (Fig. 2). Biochemical analysis of serum alkaline phosphatase, calcium and phosphorus were carried out for differential diagnosis with Paget’s disease and were shown to be within the normal limits. All clinical, radiographic and biochemical features were suggestive of the diagnosis of FCOD.

**Figure 3:**
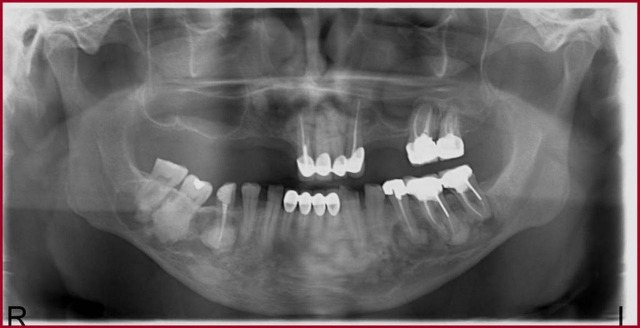
Follow-up panoramic radiograph obtained after an 30-month interval in the patient.

Because of the dense bone with poor vasculature found in FCOD, the periapical infection may progress into osteomyelitis. Therefore, the patient was motivated about the oral hygiene, referred to Prosthodontics Clinic to rehabilitate his edentulous dental arch. He has been followed up over the last 30 months and FCOD has remained asymptomatic (Fig. 3). In the period of follow-up, endodontic treatments of the upper left canine, the upper left first molar, the lower left first and second molar, the lower right second premolar with advanced caries were performed.

## Discussion

Florid cemento-osseous dysplasia was first described by Melrose et al. in 1976 (7). This condition is a reactive, non-neoplastic process that is seen most frequently in middle-aged and older women of African descent (5). The definite female gender predilection of the condition cannot be explained. In the present case, the patient was a 45-year-old Caucasian male. The cause of FCOD is unknown, and there is no good explanation for its gender and racial predilections.

FCOD may be familial with an autosomal dominant inheritance pattern, but there are only a few examples in the literature in which the familial pattern has been confirmed (7). In the present case no familial aspects of the disease could be established.

When patients are asymptomatic, conventional radiographs that exhibit multiquadrant diffuse radiopaque masses in the alveolar parts play an important role in the diagnosis. The lesions are typically found in the toothbearing areas of the jaws. The radiographic appearance, though not pathognomonic, is quite characteristic and very helpful in establishing the diagnosis (8). The use of CT has been previously reported (3). Axial CT images clearly show the location and extent of the lesion, especially in the maxilla. The expansion of the cortical bones is clearly evaluated on CT, even if it is slight. CT can be used to differentiate FCOD from lesions that exhibit a similar sclerotic appearance on conventional radiographs. In enostosis or exostosis, the high-density masses are more clearly observed on axial CT images than on occlusal radiographs, and they are found to be continuous with cortical plates (9).

Odontogenic tumors, especially cemento-ossifying fibroma, usually exhibit more buccolingual expansion than does FCOD (10).

Paget’s disease of the bone may have a cotton-wool appearance. On the other hand, this condition affects the bone of the entire mandible and exhibits loss of lamina dura, whereas FCOD is localized above the mandibular canal (9). Paget’s disease is also characterized by deformities of multiple bones and produces biochemical serum changes, such as elevated alkaline phosphatase levels (11). No biochemical alterations and other bone involvement were found in this case presented. Differential diagnosis of FCOD should also includes sclerosing osteomyelitis, which can be a complication of the disease (6,12). It appears as a single, poorly delineated opaque segment of the mandible, whereas FCOD is seen as multiple round or lobulated opaque masses. Chronic diffuse sclerosing osteomyelitis involves the body of the mandible from the alveolus to the inferior border and may extend into the ramus (13).

Once the diagnosis has been established in an asymptomatic patient, under normal circumstances, there is no need to exist further treatment. The patient should be regularly follow-up and recall examinations with prophylaxis and reinforcement of good home hygiene care to control periodontal disease and prevent tooth loss. In the absence of clinical signs, reevaluation with panoramic radiographs in every 2 or 3 years is adequate. Dental CT imaging should be considered if new symptoms or signs develop (3).

Management of the symptomatic patient is more difficult because chronic inflammation and infection develop within densely mineralized tissue. Sequestration of the cementum-like masses occurs slowly and is usually followed by healing in that area. Generally antibiotics are not effective in FCOD so their tissue diffusion is poor. Indeed biopsy increases the risk of infection or may cause jaw fractures and it is not normally justified to surgically remove these lesions as this often requires extensive surgery (1-4).

It is of major importance to realize that all dentists have a unique opportunity as well as ethical obligation to assist in the struggle against wrong dental treatments that might save patients dental health. This case report illustrates the point that periapical radiolucencies may represent benign fibro-osseous lesions that may be overlooked or result in unnecessary endodontic treatment.
